# Glucose Significantly Enhances Enterotoxigenic *Escherichia coli* Adherence to Intestinal Epithelial Cells through Its Effects on Heat-Labile Enterotoxin Production

**DOI:** 10.1371/journal.pone.0113230

**Published:** 2014-11-19

**Authors:** Prageeth Wijemanne, Rodney A. Moxley

**Affiliations:** School of Veterinary Medicine and Biomedical Sciences, University of Nebraska-Lincoln, Lincoln, Nebraska, United States of America; University of Minnesota, United States of America

## Abstract

The present study tested whether exposure of enterotoxigenic *Escherichia coli* (ETEC) to glucose at different concentrations in the media results in increased bacterial adherence to host cells through increased heat-labile enterotoxin (LT) production, thereby suggesting the effects are physiological. Porcine-origin ETEC strains grown in Casamino acid yeast extract medium containing different concentrations of glucose were washed and inoculated onto IPEC-J2 porcine intestinal epithelial cells to test for effects on adherence and host cell cAMP concentrations. Consistent with previous studies, all LT^+^ strains had higher ETEC adherence to IPEC-J2 cells than did LT^−^ strains. Adherence of the LT^−^ but not the LT^+^ strains was increased by pre-incubating the IPEC-J2 cells with LT and decreased by co-incubation with GM1 ganglioside in a dose-dependent manner (*P*<0.05). To determine whether the glucose concentration of the cell culture media has an effect on adherence, IPEC-J2 cells were inoculated with LT^+^ or LT^−^ strains in cell culture media containing a final glucose concentration of 0, 0.25, 0.5, 1.0 or 2.0%, and incubated for 4 h. Only media containing 0.25% glucose resulted in increased adherence and cAMP levels, and this was limited to IPEC-J2 cells inoculated with LT^+^ strains. This study supports the hypothesis that glucose, at a concentration optimal for LT expression, enhances bacterial adherence through the promotion of LT production. Hence, these results establish the physiological relevance of the effects of glucose on LT production and provide a basis for how glucose intake may influence the severity of ETEC infection.

## Introduction

Enterotoxigenic *Escherichia coli* (ETEC) is the most common bacterial cause of diarrhea in humans in the world [1] and an important cause of diarrhea and death in livestock, especially cattle and swine [2], [3]. ETEC cause diarrhea primarily through the effects of enterotoxins [4]. Many highly virulent ETEC isolates from swine produce F4ac (K88ac) fimbria and both heat-labile enterotoxin-I (LT) and heat-stable enterotoxin-b (STb) [5], with the genes for the two toxins linked on a large, transmissible plasmid [6].

Environmental conditions are known to affect the production of LT, heat stable enterotoxin-a (STa) and STb [7], [8], [9], [10], [11]. Glucose increases LT production through de-repression of the *eltAB* (LT) promoter [12]. Glucose suppresses synthesis of cyclic AMP (cAMP), which prevents cAMP receptor protein (CRP)-cAMP complex formation and binding to sites upstream and within the *eltA* promoter [12]. Interestingly, glucose also prevents transcription of *estB* (STb) and *estA* (STa) through catabolite repression [10], [12].

In addition to causing severe diarrhea and weight loss, LT produced by ETEC enhances the organism’s capacity to colonize the small intestines [5], [13], although this effect has not always been reproducible with isogenic LT^−^ mutant ETEC strains [14]. LT promotes adherence of ETEC to intestinal epithelial cells [15], and this effect is mediated both by enzymatic and non-enzymatic properties of the toxin [16]. Conversely, possession of *estB* has not resulted in increased colonization in inoculated 9-day-old gnotobiotic piglets [14], but instead has been associated with reduced ETEC adherence in ligated jejunal loops in fasted 5- to 8-week-old weaned pigs in association with increased fluid accumulation [17].

Mudrak and Kuehn [18] raised the hypothesis that exposure of ETEC to glucose in the proximal intestine may induce the production of LT to aid adherence and colonization of the ileum, where most ETEC cells are found in a mouse model of colonization [13]. However, to our knowledge, no studies have tested whether exposure of LT^+^ ETEC to glucose leads to increased colonization or epithelial adherence. The present study addressed the latter question, utilizing the IPEC-J2 porcine intestinal epithelial cell line and porcine-origin ETEC strains [14]. In contrast to the strains tested by Johnson et al. [15], those used in the present study included both LT^−^ and STb^−^ deletion mutants, and the LT^−^ mutants had not demonstrated reduced intestinal colonization in gnotobiotic piglets [14]. Hence, we tested the effects of LT in adherence with these strains prior to conducting experiments with glucose. In addition, we tested whether other sugars (sucrose or fructose) had an effect on LT production and secretion. We found that growth of *E. coli* in media containing glucose at a concentration optimal for LT expression enhances bacterial adherence to intestinal epithelial cells through promotion of LT production.

## Materials and Methods

### Bacterial strains and culture conditions

The following strains were used in this study: 2534-86, a wild-type (WT) porcine-origin F4ac^+^, LT^+^, STb^+^ ETEC [19]; WAM2317, a spontaneous nalidixic-acid resistant mutant of 2534-86 [19]; MUN297 (STb^−^ mutant of WAM2317); MUN298 (MUN297 complemented with STb); MUN299 (LT^−^ mutant of WAM2317); MUN300 (LT^−^ STb^−^ mutant of WAM2317); and MUN301 (MUN300 complemented with LT) [14]. DH5α (K-12 *E. coli* laboratory strain), and G58-1 (WT non-toxigenic *E. coli* strain of porcine origin [20] were also used in this study.

With the exception of experiments testing the effects of glucose and other sugars on LT secretion, strains were grown in Casamino Acid Yeast Extract (CAYE) medium containing Bacto Casamino Acids and Difco Yeast Extract at a final glucose concentration of 0.25% and pH of 8.5 [7]. Starter cultures were grown in 3.0 ml of CAYE in 15 ml tubes at 37°C and 225 rpm for 18 h. For adherence and cAMP assays, the starter culture was diluted 1∶100 in 3 ml of CAYE in 15 ml tubes and grown for 2 h at 37°C and 225 rpm.

For experiments involving the effects of different sugars on growth, pH, LT production and secretion, strains were grown in CAYE media containing glucose, sucrose or fructose at 0%, 0.25%, 0.5%, 1%, or 2% at pH 8.5. For each strain and medium, a 150-µl aliquot of starter culture was inoculated into 15 ml of the same medium in a 125-ml flask and grown at 37°C and 225 rpm for 6 h.

### IPEC-J2 cell culture conditions and bacterial adherence assays

IPEC-J2 porcine small intestinal epithelial cells [21], obtained from Dr. Thomas Burkey, University of Nebraska-Lincoln, were cultured using methods previously described [15]. Cells were cultured in Dulbecco’s modified Eagle’s medium (DMEM)-F12 medium supplemented with 5% fetal bovine serum (Sigma), 1% insulin-selenium-transferrin mix (Sigma), epidermal growth factor (5 ng/ml) (Invitrogen), 1% penicillin-streptomycin mix (Gibco) and were maintained in a humidified incubator in an atmosphere of 5% CO_2_ at 37°C. The DMEM-F12 medium supplemented with serum and other growth factors as described above contained 28 to 33 µ*M* (0.000504%–0.000594%) glucose as determined by a glucose oxidase assay (Amplex Red Glucose/Glucose Oxidase assay, Invitrogen).

IPEC-J2 cells were seeded in 24-well plates at a concentration of 10^5^ cells per well and grown to >90% confluence before inoculation. Bacterial inocula were prepared from 2-h cultures washed 3 times with PBS and seeded at a multiplicity of infection of 100∶1. Plates were incubated for 1 or 2 h in an atmosphere of 5% CO_2_ at 37°C and washed 3 times with 2 ml PBS per well (5 min per wash) to remove non-adherent bacteria. Plates were then treated with 0.25% trypsin (250 µ1 per well) at 5% CO_2_, 37°C for 5 min. The contents of each well was collected, centrifuged (2500×*g*, 5 min), re-suspended in 1 ml PBS, serially diluted, plated on LB, and incubated for 24 h at 37°C. The final CFU was measured by subtracting the CFU from control wells of the same 24-well plate lacking any IPEC-J2 cells.

To test for the effects of endogenous LT expressed by the bacteria on adherence in isogenic porcine-origin ETEC strains that had not demonstrably shown an effect of LT on colonization [14], inocula were prepared and directly applied to the IPEC-J2 cells, as described above. To test the effect of exogenous LT on adherence on these strains, LT (List Biological Laboratories) at a concentration of 1, 10, or 100 ng/ml per well was added (pre-incubation) 1 h prior to bacterial inoculation. To test the potential of GM1 ganglioside to mitigate the effects of LT on adherence, LT at a concentration of 10 ng/ml or 100 ng/ml was incubated with the cells 1 h prior to their inoculation with bacteria, and 10 ng/ml or 100 ng/ml GM1 ganglioside (Sigma) was applied to the IPEC-J2 cells at the time of bacterial inoculation. Three independent experiments were performed for each assay.

### Test of effects of different carbohydrates on LT production and secretion

To test the effects of different sugars on LT secretion, CAYE media containing glucose, sucrose or fructose at 0%, 0.25%, 0.5%, 1%, or 2% were prepared. Strains were cultured in different media as described above, with aliquots taken at 0, 2, 4, and 6 h post-inoculation (PI). These samples were centrifuged (2500×*g*, 10 min), and supernatants taken to analyze for LT concentrations by GM1-ELISA as previously described [17]. Three independent experiments were performed for each assay.

### Tests of effects of glucose on bacterial adherence and cAMP concentrations in IPEC-J2 cells

IPEC-J2 media (50 ml) were incubated with 500 µl of glucose oxidase solution with an activity of 500–2500 U/ml (MP Biomedicals) for 1.5 h at room temperature to remove glucose naturally present in the media, followed by 1 h incubation in a 65°C water bath to denature glucose oxidase. After establishing the absence of glucose by an Amplex Red Glucose/Glucose Oxidase assay (Invitrogen), glucose was added to make IPEC-J2 media with 0%, 0.25%, 0.5%, 1% and 2% glucose concentrations. IPEC-J2 cells were added to media with the varying glucose concentrations followed by bacterial inoculation and then the cells were incubated, treated, and plated in the same manner as in the bacterial adherence assays as described above, except that incubations occurred for 4 h.

To determine whether differences in glucose concentration, through their effects on the bacteria were associated with LT-mediated effects on host cells, cAMP levels of infected IPEC-J2 cells were determined. These experiments were performed using the same methods as for the experiments conducted for studying the effect of different glucose levels on adherence to IPEC-J2 cells except that instead of treating the cells with 0.25% trypsin, cells were lysed using cell lysis buffer (#9803) included in a cAMP assay kit (Cell Signaling Technology, Cyclic AMP XP Assay Kit #4339S) and tested for cAMP concentrations using this kit following the manufacturer’s instructions. Three independent experiments were performed for all assays.

### Statistical analyses

SAS Version 9.4 (Cary, NC) software was used to analyze the data for bacterial adherence assays using the least squares method with PROC GLM, with significant differences (*P*<0.05) calculated by Tukey’s method. Results for glucose effect on bacterial adherence and cAMP assay were analyzed by unpaired Student’s *t*-tests with values of *P*<0.05 considered significant.

## Results

### Confirmation that endogenously expressed LT increases adherence of porcine ETEC strains to IPEC-J2 cells

**Figure 1:**
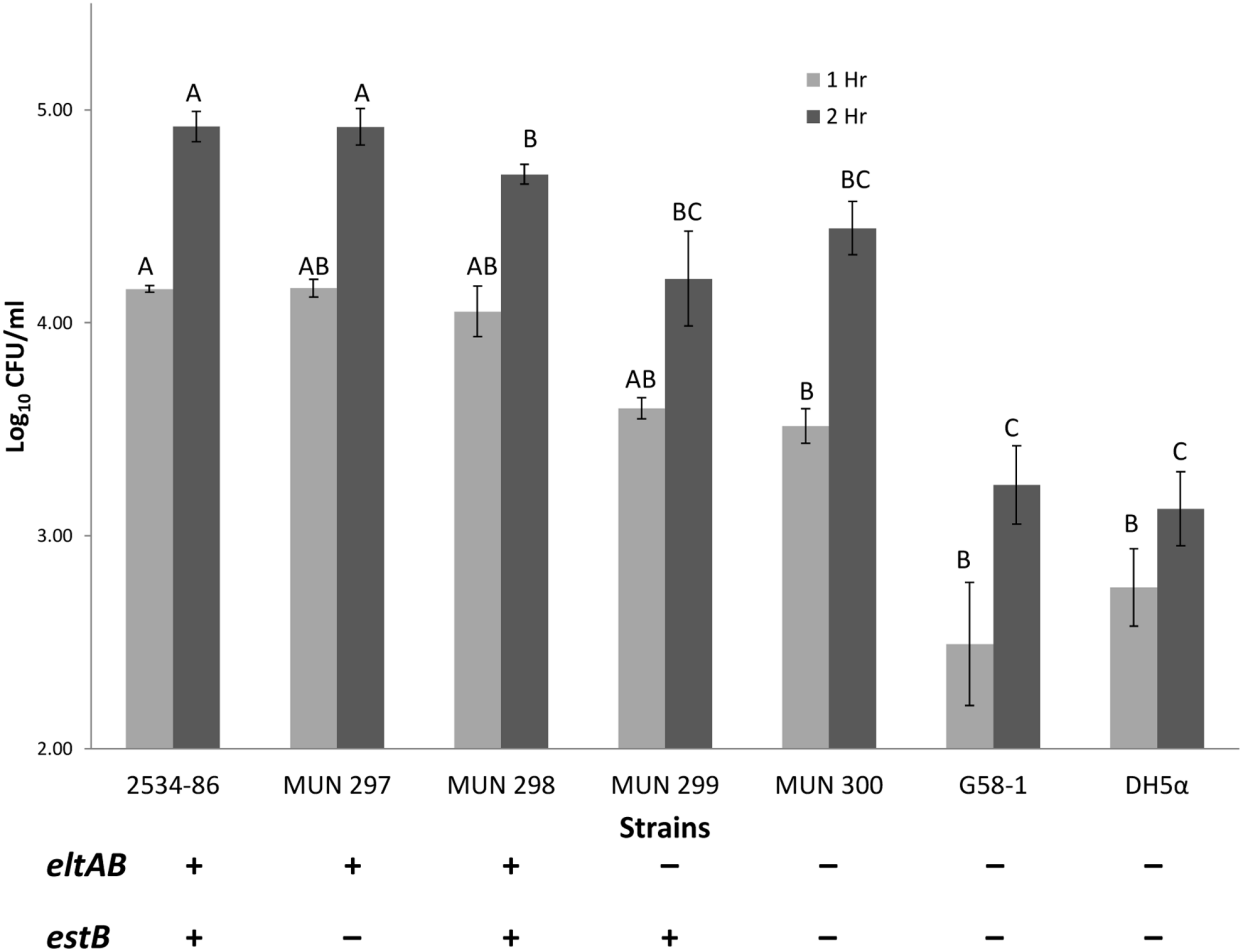
Effects of endogenous LT on adherence of isogenic porcine-origin ETEC strains to porcine intestinal epithelial (IPEC-J2) cells. Strains were cultured in Casamino Acid Yeast Extract (CAYE) medium containing 0.25% glucose, final pH 8.5, conditions which supported the production and secretion of LT. IPEC-J2 cells were inoculated at a multiplicity of infection of 100∶1 in DMEM-F12 cell culture media. Adherence of F4ac^+^ ETEC strains was compared with that of non-fimbriated LT^−^ control strains G58-1 and DH5α after 1 and 2 h of incubation. *eltAB* and *estB* denote presence (+) or absence (−) of genes for LT and STb production, respectively. Means with different letter designations indicate adherence levels that are significantly different (*P*<0.05) within the same incubation time group.

It was necessary to confirm that LT promoted the adherence of *E. coli* on IPEC-J2 cells as shown previously [15] since our studies involved a different set of isogenic strains, and ones in which the production of LT had not resulted in an increase in intestinal colonization of gnotobiotic piglets [14]. In addition, our experiments included isogenic STb^−^ mutants and STb^+^ complemented mutant strains; the latter had reduced adherence in ligated jejunal loops in weaned pigs in association with fluid accumulation [17]. At 1 h PI, 2534-86 (WT LT^+^ STb^+^) had significantly greater adherence than non-isogenic LT^−^ STb^−^ controls, G58-1 and DH5α (*P*<0.05; Fig. 1). At 2 h PI, 2534-86 and MUN297 (LT^+^ STb^−^) had significantly greater adherence than LT^−^ strains and MUN298 (LT^+^ STb-complemented mutant; *P*<0.05; Fig. 1). These results confirmed the conclusions of Johnson et al. [15] that endogenously expressed LT promotes adherence of porcine-origin ETEC to porcine intestinal epithelium.

### Demonstration of dose-dependent increase by exogenous LT and inhibition by GM1 ganglioside of adherence of ETEC to IPEC-J2 cells

**Figure 2:**
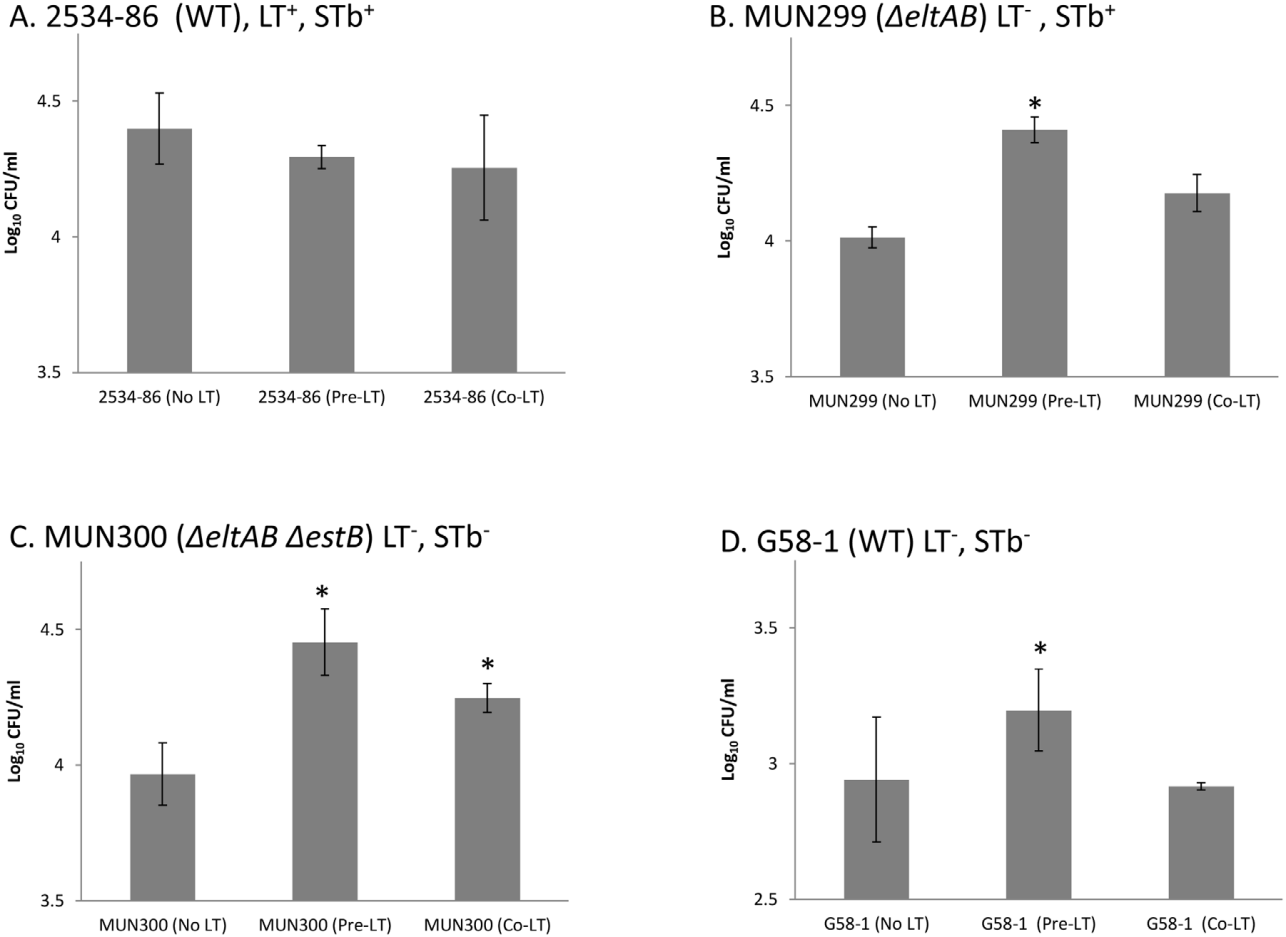
Effects of exogenous LT on adherence of isogenic porcine-origin ETEC strains to porcine epithelial (IPEC-J2) cells. A. Wild-type LT^+^ STb^+^ strain 2534-86. B. LT^−^ (Δ*eltAB*) strain MUN299. C. LT^−^ STb^−^ (Δ*eltAB* Δ*estB*) strain MUN300. D. Wild-type non-enterotoxigenic strain G58-1. IPEC-J2 cells were incubated 1 h prior to inoculation (Pre-LT) or co-incubated at the time of inoculation (Co-LT) with 100 ng/ml LT. Asterisk (*) denotes adherence levels of a given LT treatment were significantly different (*P*<0.05) from that of the non-LT-treated control (No LT).

**Figure 3:**
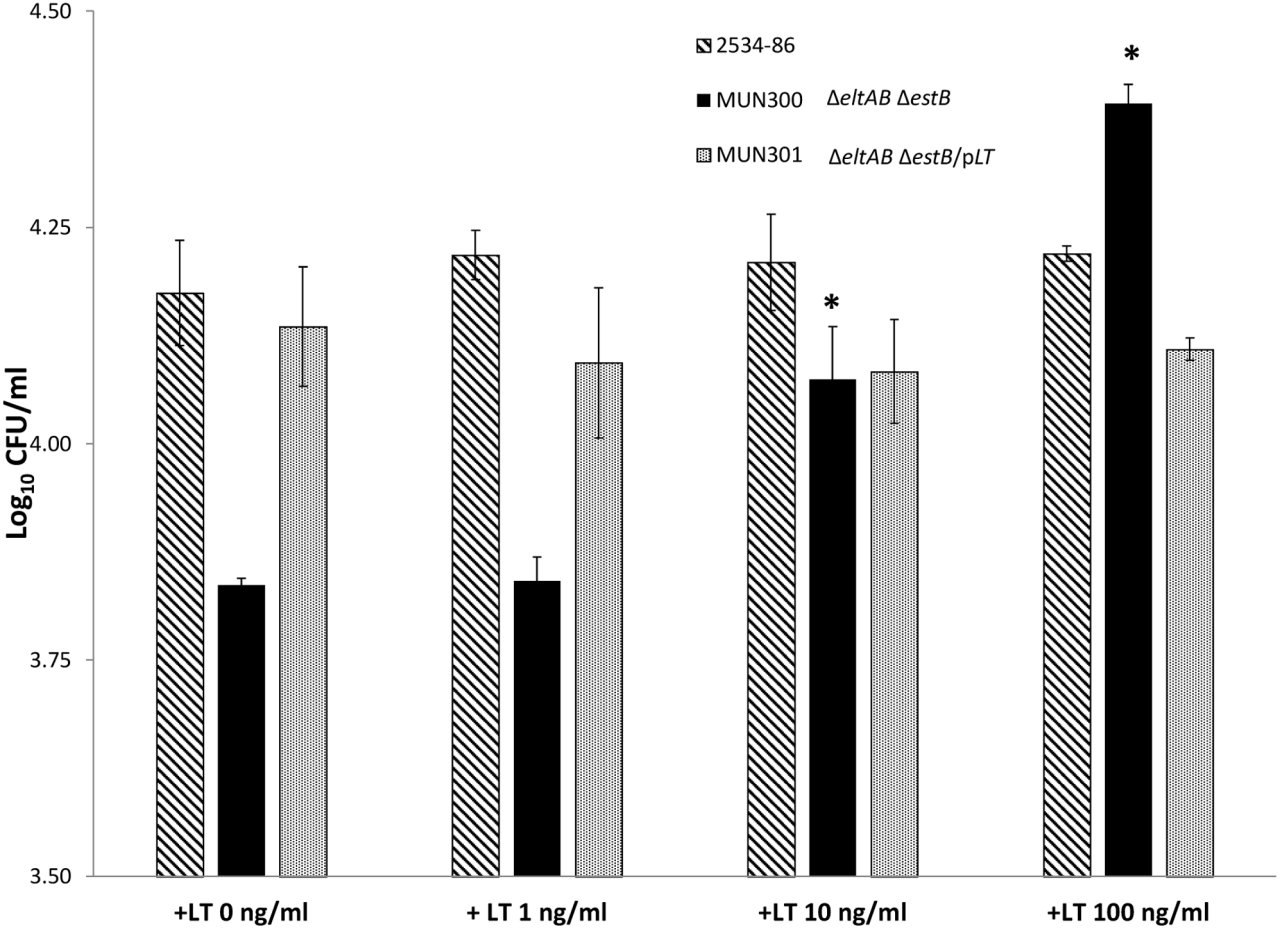
Dose-dependent effect of exogenous LT on the adherence of porcine-origin ETEC strains to porcine epithelial (IPEC-J2) cells. IPEC-J2 cells were pre-treated with 1, 10 or 100 ng/ml of exogenous LT 1 h prior to inoculation with wild-type LT^+^ STb^+^ strain 2534-86, LT^−^ STb^−^ (Δ*eltAB* Δ*estB*) MUN300, or LT^+^ complemented (Δ*eltAB* Δ*estB/*p*eltAB*) strain MUN301. Asterisk (*) denotes a level of adherence significantly different (*P*<0.05) from that of the same strain without exogenous LT.

**Figure 4:**
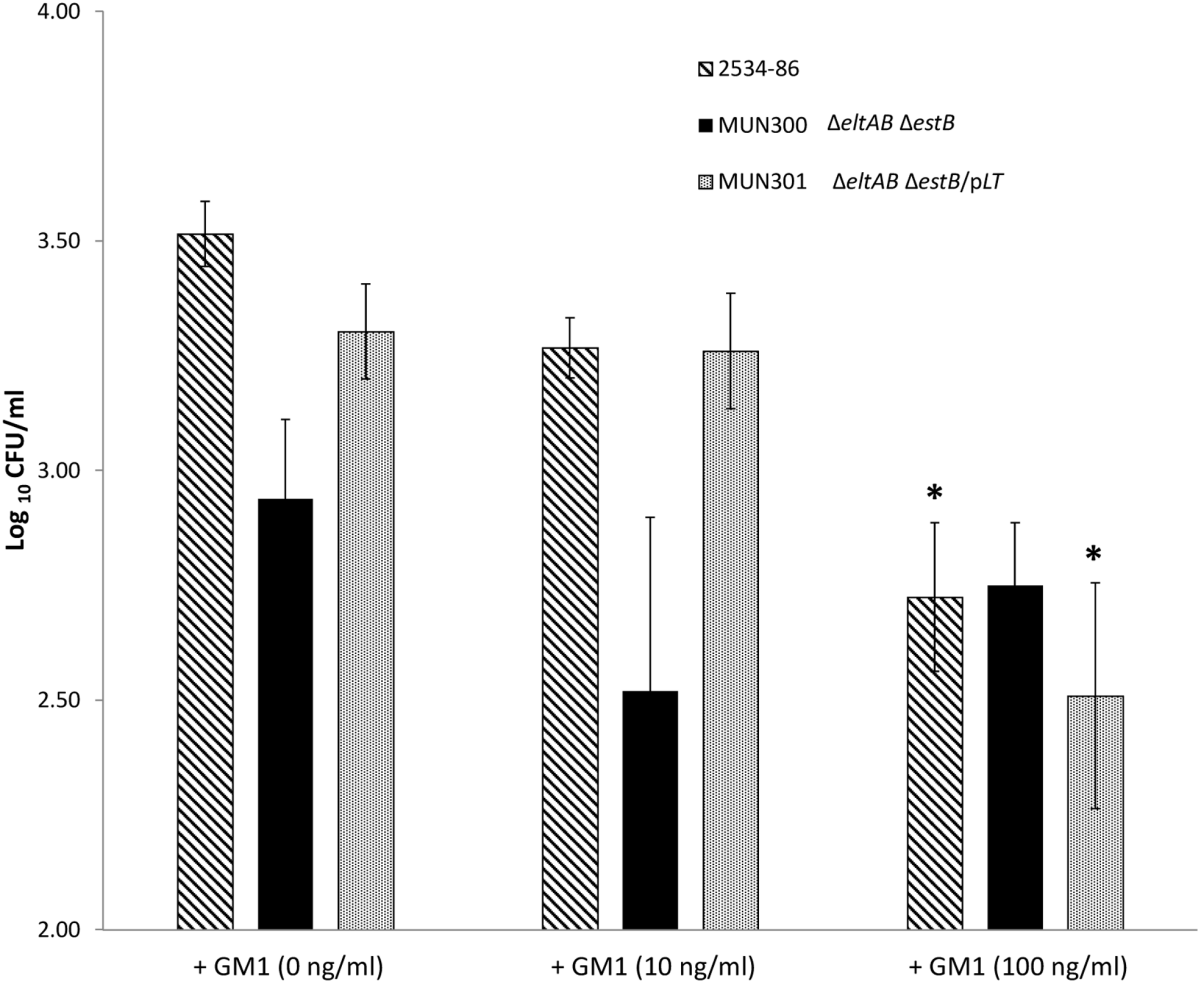
Inhibitory dose-response of exogenous GM1 ganglioside on the adherence of porcine-origin ETEC strains to porcine epithelial (IPEC-J2) cells. IPEC-J2 cells were treated with 10 or 100 ng/ml of GM1 at the time of inoculation with wild-type LT^+^ STb^+^ strain 2534-86, LT^−^ STb^−^ (Δ*eltAB* Δ*estB*) MUN300, or LT^+^ complemented (Δ*eltAB* Δ*estB/*p*eltAB*) strain MUN301. Asterisk (*) denotes a level of adherence significantly different (*P*<0.05) from that of the same strain without exogenous GM1.

To confirm the results of Johnson et al. [15] that adherence of LT^−^ porcine *E. coli* strains could be increased by exogenously applied LT, IPEC-J2 cells were incubated with 100 ng/ml of LT, either 1 h prior to or at the time of inoculation. Addition of LT at either time-point did not affect the adherence level of 2534-86 (Fig. 2A). However, the adherence levels of MUN299 (LT^−^ STb^+^) Fig. 2B), MUN300 (LT^−^ STb^−^; Fig. 2C) and G58-1 (LT^−^ STb^−^; Fig. 2D) were significantly increased (*P*<0.05) when the IPEC-J2 cells were pre-incubated with exogenous LT. Only the adherence of MUN300 was significantly increased (*P*<0.05) when IPEC-J2 cells were co-incubated with exogenous LT. Pre-incubation with 10 and 100 but not 1 ng/ml LT resulted in a significant increase in adherence, demonstrating that the effect was dose-dependent (Fig. 3). Addition of GM1 at 100 but not 10 ng/ml at the time of inoculation significantly reduced adherence of 2534-86 and MUN301 (LT^+^ STb^−^; Fig. 4). The adherence of MUN300 was not reduced by the addition of 10 or 100 ng/ml GM1 (Fig. 4). These results confirmed the findings of Johnson et al. [15] that exogenous LT increases the adherence of LT^−^ *E. coli* and extended these results by demonstrating that the pro-adherent effects of LT are dose-dependent. In addition, dose-dependent inhibition of adherence by GM1 ganglioside was demonstrated.

### Effect of different concentrations of glucose and other sugars on LT production

**Figure 5:**
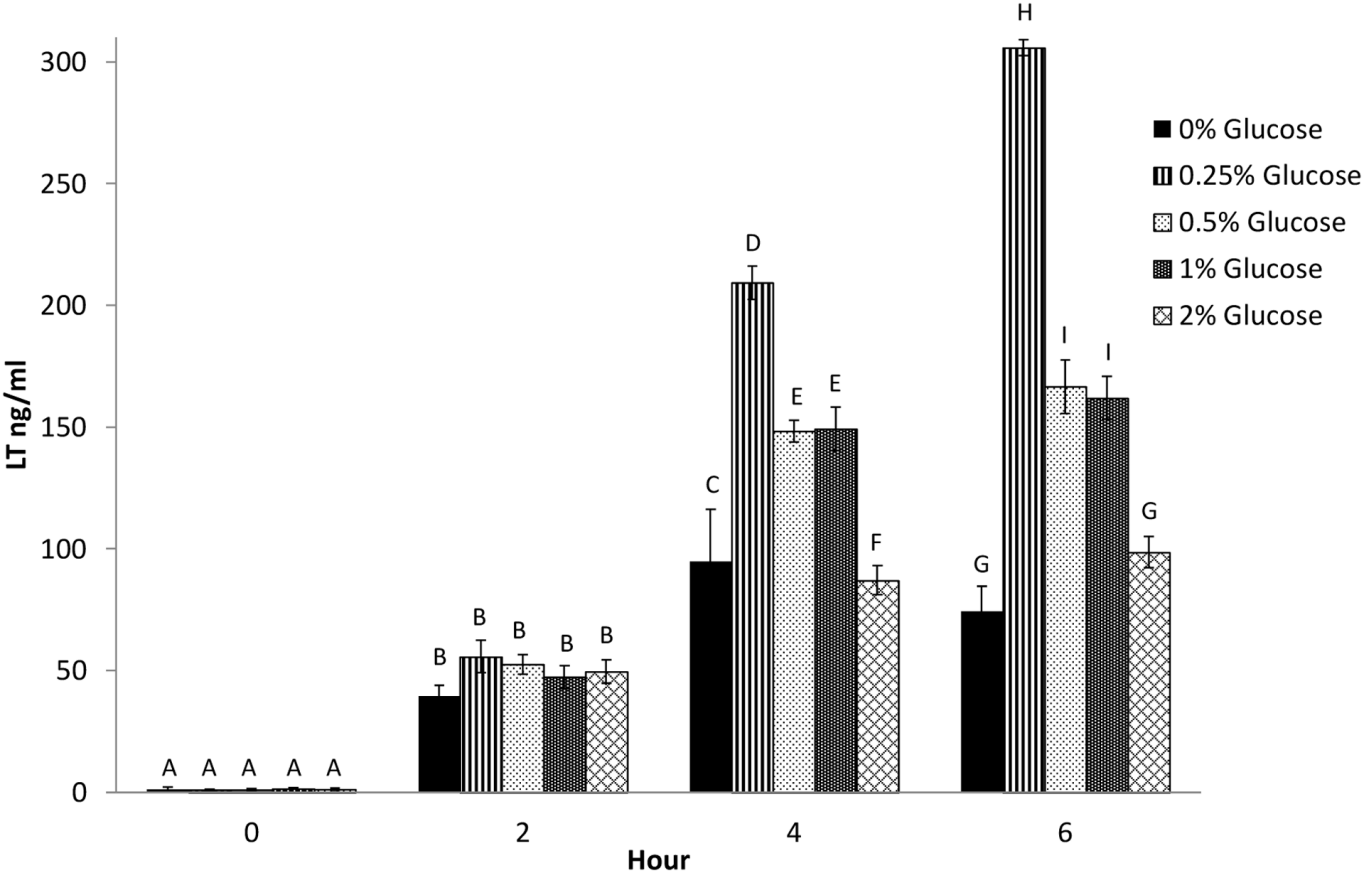
Effect of glucose concentration of the media on LT production by wild-type LT^+^ STb^+^ strain 2534-86. CAYE media with glucose concentrations of 0, 0.25, 0.5, 1 and 2% adjusted to pH 8.5 were inoculated with an overnight (18 h) culture of 2534-86 and LT concentrations in the culture supernatants were measured at 0, 2, 4 and 6 h of culture by GM1-ELISA. Separate Tukey’s tests for multiple means comparisons of LT secreted for different glucose concentrations at each time point were conducted. Different letter designations denote mean LT concentrations that are significantly different within a sampling time point (*P*<0.05).

**Figure 6:**
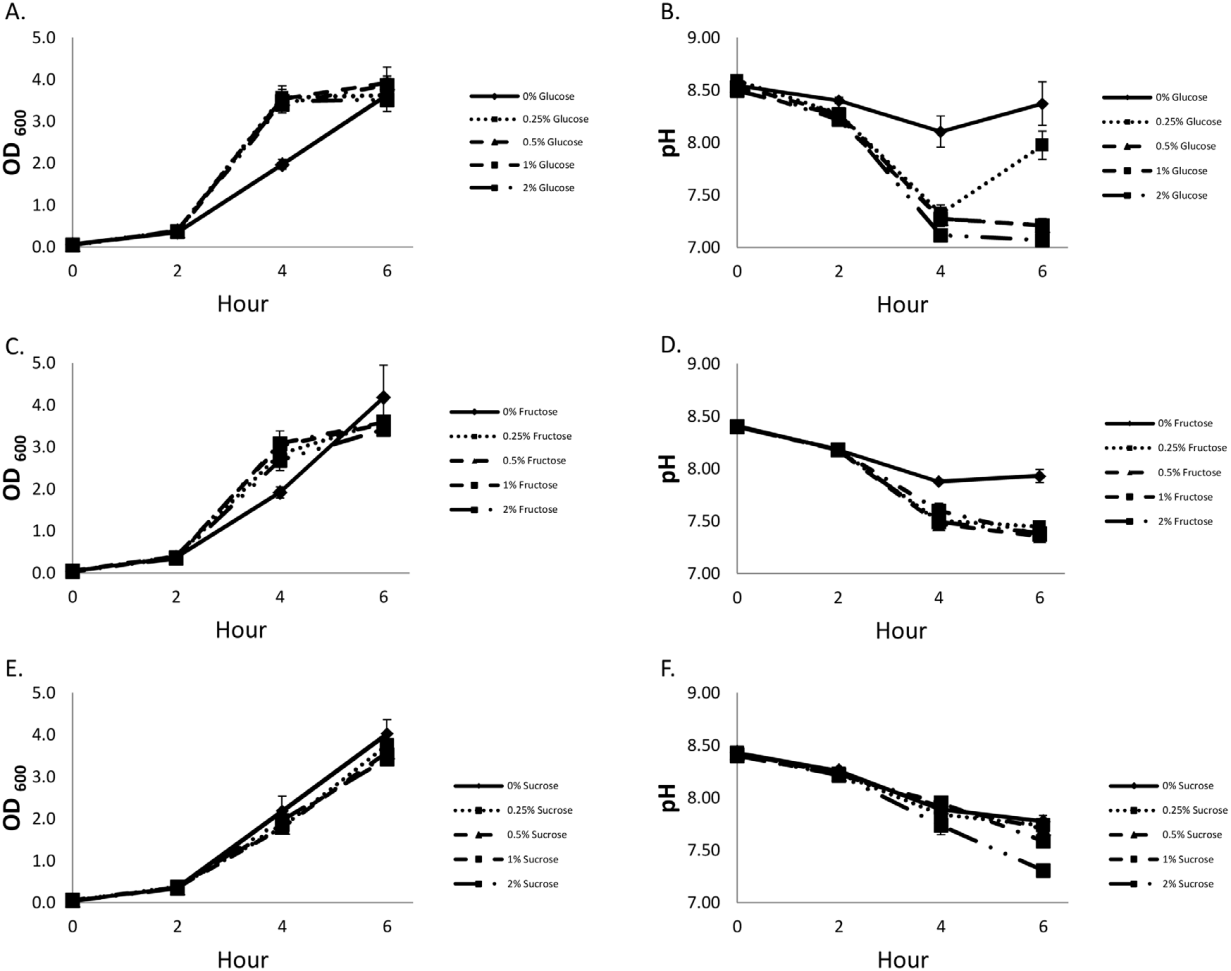
Growth and pH of wild-type LT^+^ STb^+^ strain 2534-86 in CAYE medium containing glucose (A, B), fructose (C, D), or sucrose (E, F) at concentrations of 0, 0.25, 0.5, 1 and 2%. CAYE media, pH 8.5, containing different carbohydrate sources were inoculated with an overnight (18 h) culture of 2534-86, and incubated at 37°C and 225 rpm for 6 h. Growth (OD_600_) and pH were measured on samples collected at 0, 2, 4 and 6 h of culture.

In studies involving H10407 [7] and other human-origin strains [11], the optimal glucose concentration for LT production in CAYE medium was 0.25%. Kunkel and Robertson [9] reported that LT levels increase as the pH is raised in 0.5 U intervals from 6.0 to 10.0, with LT primarily remaining cell-bound at a pH lower than 7.0 and most LT released between pH 7.5 and 8.0. To determine the effects of glucose concentration on secreted LT by 2534-86, this strain was grown in CAYE medium containing 0, 0.25, 0.5, 1.0 or 2.0% glucose, supernatants were sampled at 0, 2, 4 and 6 h of culture, and growth and pH were monitored. The LT concentrations in the culture supernatants increased with time in all glucose concentrations; however, consistent with human-origin strains, the medium containing 0.25% glucose yielded the highest LT concentration at 4 and 6 h of culture (*P*<0.05; Fig. 5). No significant differences in cell growth were detected in media containing 0.25 to 2.0% glucose (Fig. 6A); however, the pH of media containing 0.5 to 2.0% glucose was significantly lower than that of media containing 0 and 0.25% glucose at 6 h of culture (Fig. 6B). These results were consistent with previous reports [8], [9] that glucose at a concentration of 0.25% yielded maximal LT production, and decreases in the pH from 8.0 to 7.0 were associated with a significant decrease in the LT concentration in the culture supernatant (Fig. 5).

**Figure 7:**
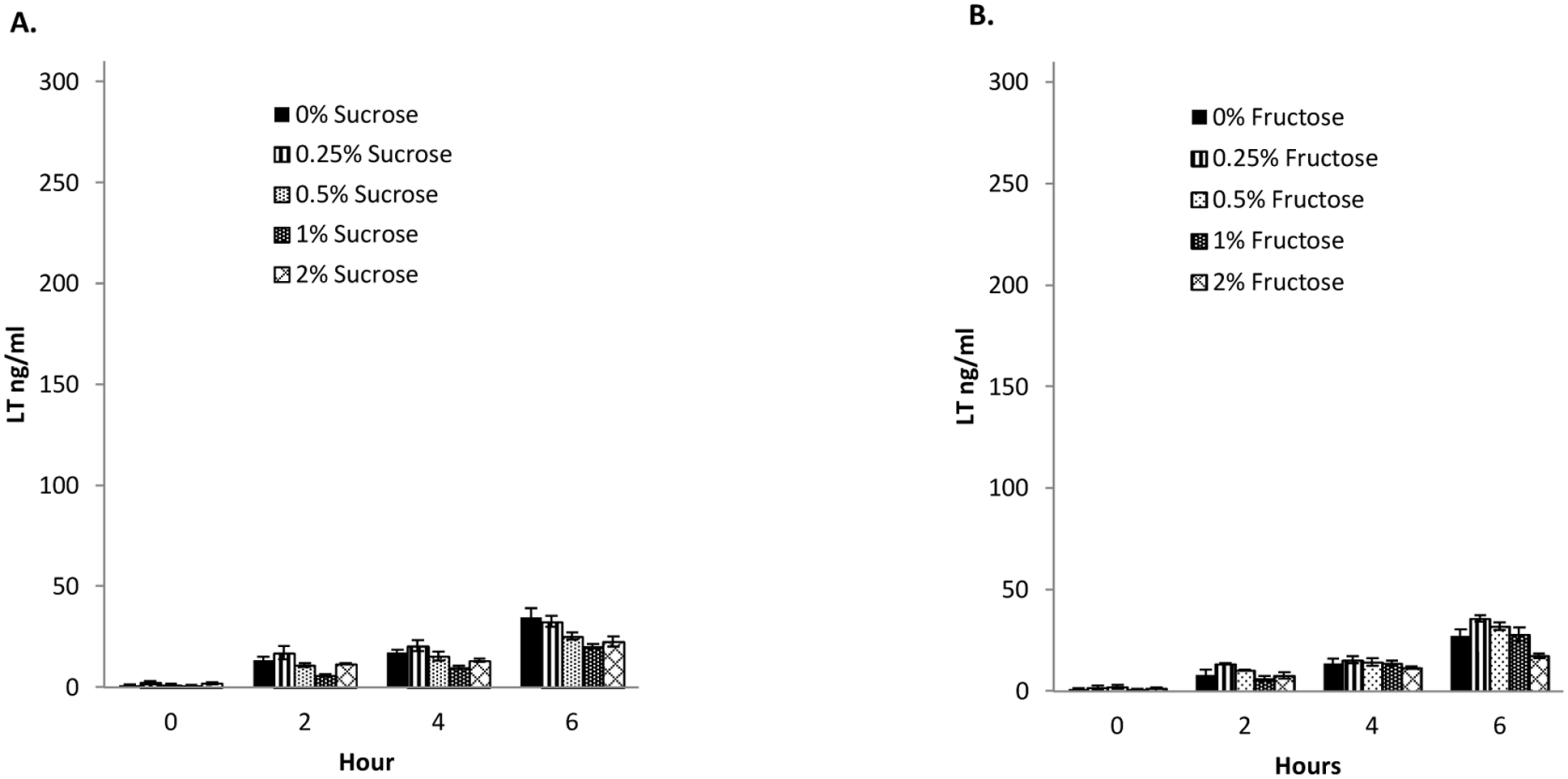
Effect of sucrose (A) and fructose (B) in the culture medium on LT production and secretion by wild-type LT^+^ STb^+^ strain 2534-86. CAYE media with fructose or sucrose at concentrations of 0, 0.25, 0.5, 1 and 2% were inoculated with an overnight (18 h) culture of 2534-86 and LT concentrations in samples of culture supernatant collected at 0, 2, 4 and 6 h of culture were determined by GM1-ELISA.

Hedge et al. [11] reported that osmotic stress (induced by NaCl supplementation to CAYE medium) reduces LT production. To test whether strain 2534-86 grown in media containing glucose at concentrations higher than 0.25% was under osmotic stress that may have affected LT production, we repeated the experiments with sucrose and fructose in place of glucose. The levels of LT produced with different concentrations of sucrose or fructose were several folds lower than that in the glucose counterpart (Fig. 7). Furthermore, neither 0.25% sucrose nor fructose yielded a higher LT concentration compared to other concentrations of the same sugar, in contrast to what had been seen with glucose. Growth curves in CAYE medium containing different concentrations of sucrose and fructose revealed minor reductions in growth rate (mainly sucrose) and pH decline compared to their glucose counterparts (Fig. 6C–F). Collectively, these results tended to rule out osmotic stress as the cause of suboptimal LT production at higher glucose concentrations. Since glucose and fructose share the same molecular weight the osmotic pressures exerted by them are identical. In media containing glucose and fructose at 0.25, 0.5, 1.0 and 2.0% the osmotic pressures calculated by the Van’t Hoff equation were 0.34, 0.68, 1.36 and 2.71 atm, respectively; in the case of sucrose, they were 0.18, 0.36, 0.71, and 1.43 atm. Therefore, the lower LT production occurring at glucose concentrations higher than 0.25% was not the result of differences in osmotic pressure.

### Effect of glucose on ETEC adherence to IPEC-J2 cells

**Figure 8:**
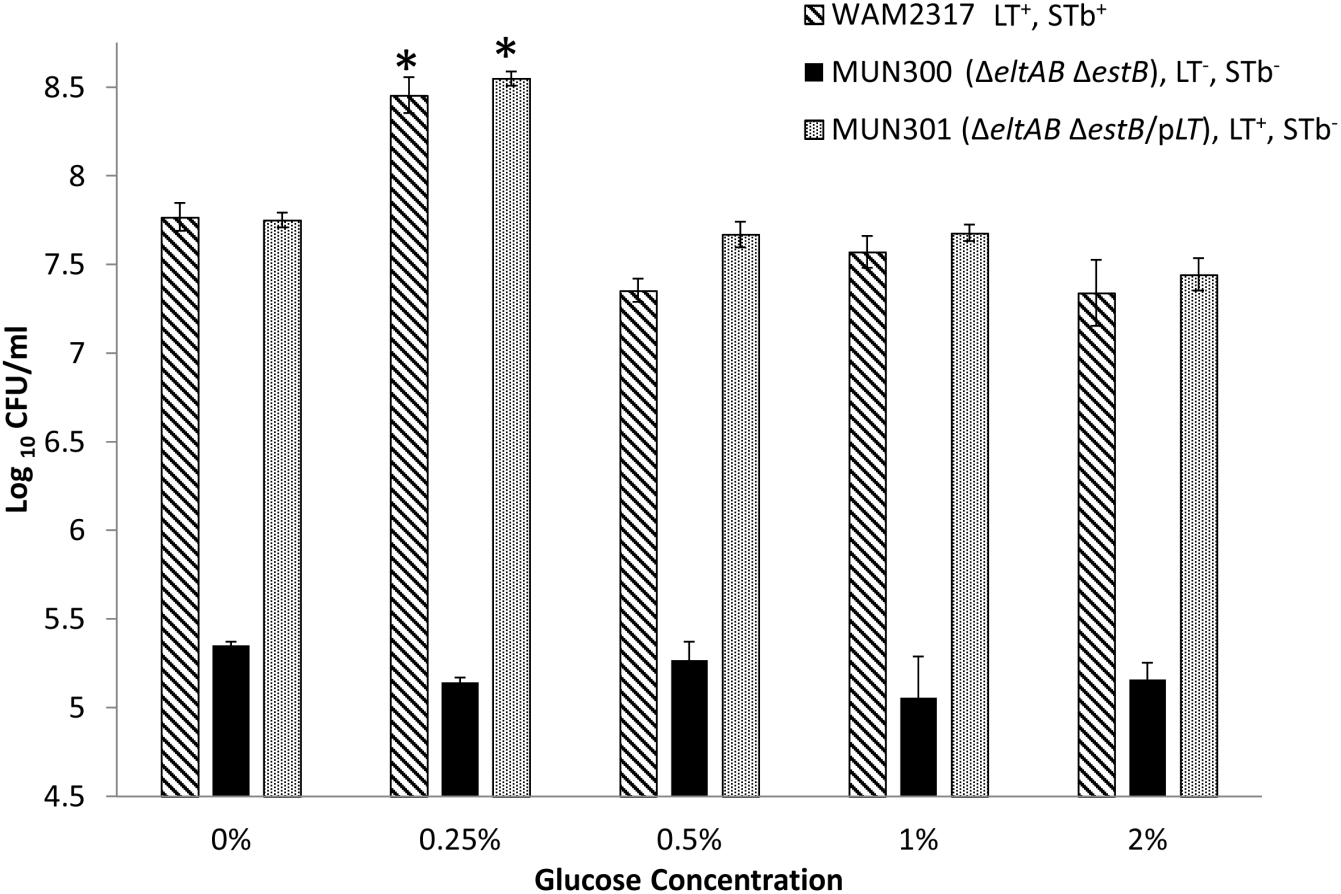
Effect of glucose in the IPEC-J2 cell culture media on adherence of porcine ETEC strains to porcine epithelial (IPEC-J2) cells. IPEC-J2 cells in the presence of DMEM-F12 media containing 0, 0.25, 0.5, 1 and 2% glucose were inoculated with a 2 h culture of strain WAM2137 (spontaneous nalidixic acid-resistant mutant derivative of wild-type LT^+^ STb ^+^2534-86), MUN300 [LT^−^ STb^−^ (Δ*eltAB* Δ*estB*) derivative of WAM2317], or MUN301 (LT^+^ complemented derivative of strain MUN300) at a multiplicity of infection of 100∶1 and incubated for 4 h. Asterisk (*) denotes a level of adherence significantly different (*P*<0.05) from that of the same strain in IPEC-J2 cell culture media containing other glucose concentrations.

**Figure 9:**
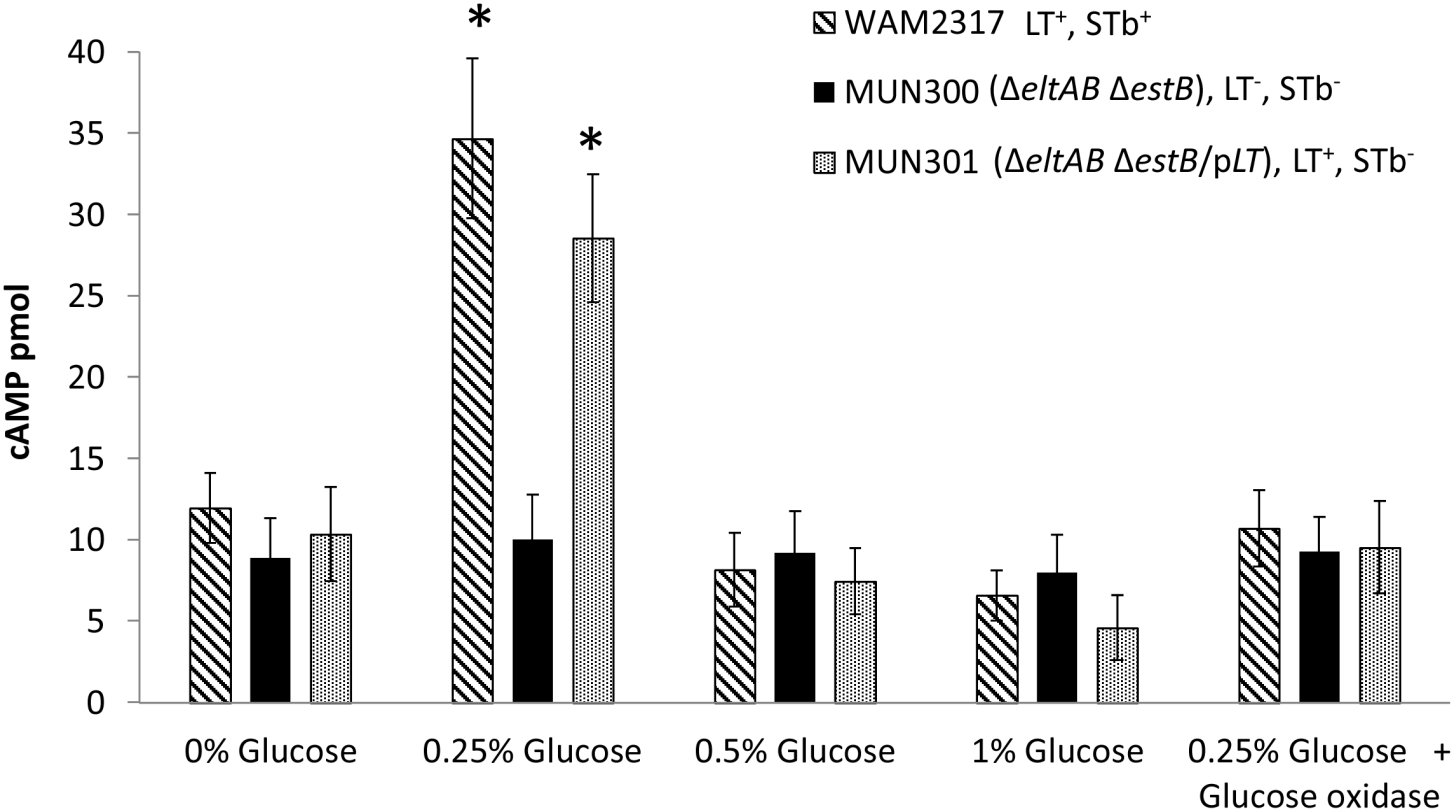
Effect of glucose in the IPEC-J2 cell culture media on cAMP levels of IPEC-J2 cells infected with porcine ETEC strains. IPEC-J2 cells in the presence of DMEM-F12 media containing 0, 0.25, 0.5, 1% glucose and 0.25% glucose + glucose oxidase were inoculated with a 2 h culture of the strains at a multiplicity of infection of 100∶1 and incubated for 4 h. Strains included WAM2137 (spontaneous nalidixic acid-resistant mutant derivative of wild-type LT^+^ STb^+^2534-86), MUN300 [LT^−^ STb^−^ (Δ*eltAB* Δ*estB*) derivative of WAM2317], or MUN301 (LT^+^ complemented derivative of strain MUN300). Asterisk (*) denotes a level of adherence significantly different (*P*<0.05) from that of the same strain grown in other glucose concentrations.

To determine whether the glucose concentration of the cell culture media has an effect on adherence of ETEC to porcine epithelial cells through induction of increased LT production and secretion, IPEC-J2 cells were inoculated with WAM2317 (spontaneous Nal^R^ mutant of 2534-86), MUN300, or MUN301 in cell culture media (DMEM-F12) containing a final glucose concentration of 0, 0.25, 0.5, 1.0 or 2.0%, and incubated for 4 h. Only media containing 0.25% glucose resulted in an increased adherence level, and this was limited to WAM2317 and MUN301 (Fig. 8). MUN300 did not show any difference in adherence level at any glucose concentration. To determine whether the increased adherence level was associated with the toxigenic effects of LT, the same experiment was performed and the infected IPEC-J2 cells were lysed to assess the intracellular cAMP levels. Only IPEC-J2 cells infected in the presence of 0.25% glucose showed higher cAMP levels for WAM2317 and MUN301 (Fig. 9). MUN300 did not show any increase in the cAMP levels at any glucose concentration.

## Discussion

This study demonstrated that exposure of LT^+^ ETEC bacteria to glucose at a concentration optimal for LT production and secretion significantly increases bacterial adherence to intestinal epithelial cells through the effects of LT. This hypothesis had been raised by other investigators [18], but not tested. Our work also supports the conclusions of Johnson et al. [15] that both endogenously expressed and exogenously applied LT enhance the adherence of *E. coli* to intestinal epithelial cells. A recent study by Fekete et al. [16] using other porcine strains also found that LT expression enhanced adherence. However, in contrast to these studies, our experiments included isogenic STb^−^ deletion and complemented mutants, and strains in which expression of LT did not significantly increase intestinal colonization of gnotobiotic piglets [14]. The lack of a detectable increase in colonization raised the question whether LT expression or complementation would increase their capacity for epithelial adherence. The present study confirmed that LT expression increases the epithelial cell adherence capacity of these strains. We hypothesize that the susceptibility of some litters of F4ac (K88) receptor-positive piglets to colonization by F4ac ETEC may be so great as to mask the pro-adherence effects of LT.

The present study confirmed the important role of glucose in LT production and control of pH in LT secretion or release from the bacterial cell. Delivery of LT to the host epithelial cells requires that the toxin first be secreted from the bacterium, a process known to require a type II secretion system (T2SS) [22]. In order to deliver LT to the host cell, the bacterium localizes the T2SS and LT secretion in polarized fashion with transfer of the preformed toxin at the site of contact of the bacterium with the host cell surface [23]. LT also has been shown to be delivered to host epithelial cells via outer membrane vesicles, to which it has coated [24], [25], [26].

The molecular mechanisms by which LT promotes bacterial adherence are not fully understood, but evidently involve the effects of A subunit-mediated intoxication and bacterial sensing of host cell-derived cAMP. Johnson et al. [15] demonstrated that an inhibitor of protein kinase A abrogated LT’s ability to promote subsequent bacterial adherence, and that increased adherence was not due to changes in the surface expression of the host receptor for F4ac fimbrial adhesin. Through the testing of LT A-subunit mutants, the authors further demonstrated that ADP-ribosylation activity was necessary to effect changes in bacterial adherence. Pre-treatment of WT non-enterotoxigenic strain G58-1 with cAMP significantly increased its capacity to adhere to IPEC-J2 cells. De novo bacterial protein synthesis appeared to be required, as treatment of 2534-86 with tetracycline reduced the ability of cAMP to promote subsequent adherence. Incubation of 2534-86 but not 2534-86Δ*eltAB* with supernatants of IPEC-J2 cells intoxicated with LT increased expression of FaeG, the major F4ac fimbrial subunit; however, IPEC-J2 cells intoxicated with LT did not increase expression of the F4ac fimbrial receptor. It is possible that LT intoxication may induce expression of a receptor for a heretofore unrecognized adhesin.

Fekete et al. [16] reported that the pro-adherence effects of LT are due to a combination of enzymatic and non-enzymatic properties. These conclusions were based on experiments in which adherence was unaltered by cycloheximide treatment which prevented host cell protein synthesis. Pretreatment of IPEC-J2 cells with LT promoted adherence of negatively charged latex beads (a surrogate for bacteria which carry a negative charge), and this effect was inhibited by cycloheximide, suggesting LT may induce a change in epithelial cell membrane potential. These authors concluded that LT may enhance ETEC adherence by promoting an association between the LT B-subunit and epithelial cells, and by altering the surface charge of the host plasma membrane. Collectively, this study and the one by Johnson et al. [15] suggest that different molecular mechanisms may be involved in the LT-induced promotion of bacterial adherence.

ETEC is an important cause of neonatal and post-weaning diarrhea in swine, and diet has long been recognized to have a significant effect on enteric colibacillosis in recently-, and especially, early-weaned pigs. High starch (e.g., cereal meal) and low indigestible fiber intake increase and low starch and high indigestible fiber intake reduce ETEC-mediated diarrheal disease in weanling pigs with ETEC colonizing the proximal small intestine [27]. Replacing 5% of ground corn with glucose in the feed of recently weaned baby pigs further increases the incidence of diarrhea [28]. Feeds containing viscous soluble fiber (given to promote development of the large intestine) stimulate proliferation of ETEC in newly-weaned pigs, an effect thought to result from a decreased rate of passage of digesta through the small intestine, and interference with digestion and absorption of nutrients [29], [30]. To our knowledge, the only hypothesis that has been put forth for the exacerbation of ETEC-mediated disease by excess glucose in the intestine is that it promotes increased bacterial growth.

ETEC isolated from cases of post-weaning colibacillosis in swine almost exclusively express F4 or F18 fimbria, with 100 or 39.5% of these isolates, respectively, producing LT [31]. Porcine ETEC that express F4ac fimbria in particular, effectively colonize the duodenum as well as more distal portions of the small intestine [32]. The present study demonstrates that glucose, at an optimal concentration, promotes bacterial adherence to the intestinal epithelium through the increased production of LT. We propose that exposure of LT^+^ ETEC to glucose in conjunction with bicarbonate buffering in the duodenum [33], promotes the expression and secretion of LT. LT, through induction of cAMP release by intoxicated host cells, may induce expression of F4ac fimbria by ETEC on the host cell surface [15] and also enhance ETEC adherence to the intestinal epithelium prior to fimbrial expression through alteration of the host cell membrane potential [16], thereby initiating colonization in the proximal small intestine.

## Conclusion

Exposure of enterotoxigenic *Escherichia coli* (ETEC) to glucose at 0.25% resulted in significantly increased adherence of these bacteria to enterocytes by inducing production and secretion of heat-labile enterotoxin (LT). These results establish the physiological relevance of the effects of glucose on LT production, and provide an additional hypothesis beyond that of increased bacterial growth to explain how dietary glucose may increase the severity of ETEC infections.
